# Quantitative Association between Computed-Tomography-Based L1 Skeletal Muscle Indices and Major Adverse Clinical Events Following Percutaneous Coronary Intervention

**DOI:** 10.3390/jcm12237483

**Published:** 2023-12-03

**Authors:** Eun Jin Park, So Yeon Park, Jaeho Kang, Wonsang Chu, Dong Oh Kang

**Affiliations:** 1 Cardiovascular Center, Department of Internal Medicine, Korea University Guro Hospital, Korea University College of Medicine, Seoul 08308, Republic of Korea; 2 Health Promotion Center, Department of Radiology, Gangnam Severance Hospital, Yonsei University College of Medicine, Seoul 06273, Republic of Korea

**Keywords:** computed tomography, coronary artery disease, percutaneous coronary intervention, prognosis, sarcopenia, skeletal muscle index

## Abstract

Sarcopenia is as a non-traditional risk factor for atherosclerotic cardiovascular disease. Further investigation is required to elucidate the prognostic significance of computed tomography (CT)-based sarcopenia assessment in coronary artery disease (CAD). We prospectively enrolled 475 patients, who underwent coronary stent implantation and peri-procedural CT scans within one month. Skeletal muscle index (SMI) was assessed cross-sectionally at the first lumbar vertebra (L1) level. The participants were grouped based on sex-specific L1 SMI quartiles. The primary endpoint was all-cause mortality, and the secondary composite endpoint was major adverse cardiovascular events (MACEs) over a 3-year follow-up period. Three-year all-cause mortality and MACE incidence increased significantly in patients in the lower L1 SMI quartiles compared to those of patients in the higher quartiles (*p* < 0.001). The individual composite endpoints consistently showed a higher incidence in the lower quartiles of L1 SMI (*p* < 0.001). In multivariable analysis, the lower L1 SMI quartiles independently predicted 3-year all-cause mortality and MACEs (lowest vs. highest quartiles, respectively: OR 4.90 (95% CI 1.54–15.5), *p* = 0.007; and OR 12.3 (95% CI 4.99–30.4), *p* < 0.001). In conclusion, CT-based L1 SMI demonstrated a distinct dose-dependent relationship with future MACEs in CAD patients undergoing percutaneous coronary intervention, thereby enhancing cardiovascular risk stratification.

## 1. Introduction

Sarcopenia has gained recognition as a prominent indicator of both frailty and the aging process, often coinciding with functional and metabolic impairments [1,2]. This condition is characterized by an age-related decrease in skeletal muscle strength and mass [3,4]. Recent international guidelines and expert consensus documents have defined sarcopenia as a reduction in muscle strength and skeletal muscle mass, with values falling below two standard deviations from the mean of healthy reference subjects [5,6,7,8]. Previous studies have suggested that sarcopenia may serve as a potential predictor of the occurrence and clinical outcomes of atherosclerotic cardiovascular disease (ASCVD) [9,10,11]. Among the various methods available for assessing skeletal muscle mass, computed tomography (CT) is the standardized approach for precise measurement [6,7,8,11]. CT-based assessment of skeletal muscle mass was recently validated across a diverse spectrum of ASCVD conditions, including individuals undergoing surgical or interventional treatment for severe aortic stenosis [12,13,14], abdominal aortic aneurysm [15,16], peripheral artery disease [17,18,19], and coronary artery disease (CAD) [20,21]. However, despite its potential to offer valuable prognostic insights beyond traditional risk factors, the clinical application of CT-based skeletal muscle mass assessment is frequently hindered by variations in measurement protocols and diagnostic threshold values employed in different studies. Furthermore, the definition of sarcopenia based on specific diagnostic cut-offs may limit the accurate risk assessment of individuals near these thresholds and impede the establishment of a clear dose-dependent relationship between skeletal muscle mass and future cardiovascular outcomes. Exploring this quantitative relationship has the potential to broaden its clinical implications by improving risk stratification to effectively modulate residual risk in CAD patients under optimal medical therapy.

Recently, our group demonstrated the prognostic significance of identifying low skeletal muscle mass using CT in patients who underwent successful coronary stent implantation [20]. Moreover, we introduced sex-specific diagnostic threshold values at the first lumbar vertebral (L1) level that are specifically applicable to the East Asian population. However, despite these advancements, questions remain regarding the quantitative relationship between skeletal muscle mass and observed cardiovascular outcomes as well as whether this prognostic impact differs across short- and long-term follow ups. Based on our previous findings, we hypothesized that the CT-based assessment of skeletal muscle mass at the L1 level may exhibit a dose-dependent relationship with future cardiovascular outcomes. Furthermore, we anticipated that this relationship would persist over both short- and long-term follow-ups. To address this issue, we conducted an extended quantitative comparative subgroup analysis of our previous cohort via stratification into sex-specific quartiles based on their CT-derived L1 skeletal muscle indices (SMIs) and performed landmark analysis at the one-year follow up.

## 2. Materials and Methods

### 2.1. Study Population and Enrollment

**Figure 1:**
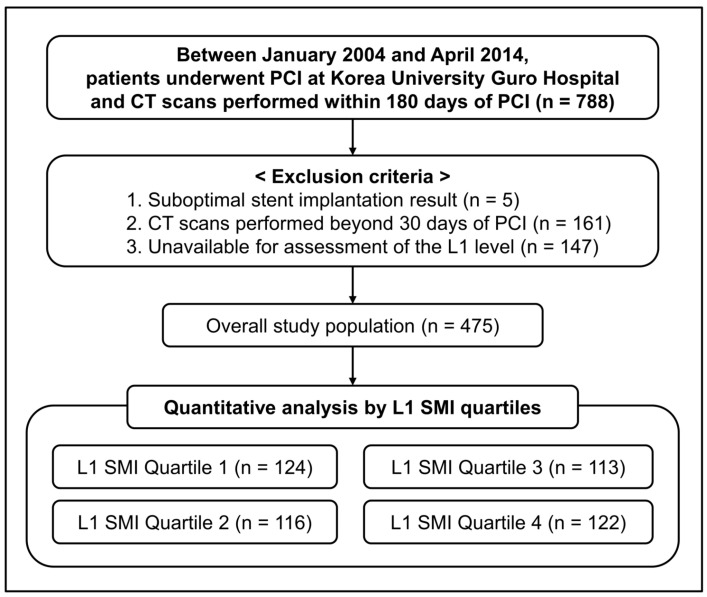
Overall study flowchart. CT, computed tomography; L1, first lumbar vertebra; PCI, percutaneous coronary intervention; SMI, skeletal muscle index.

The study population was prospectively enrolled from the percutaneous coronary intervention (PCI) registry at Korea University Guro Hospital (Seoul, Republic of Korea) between January 2004 and April 2014. Data were analyzed retrospectively. The present study was newly conducted as an extended comparative subgroup analysis derived from a previous cohort [20]. Briefly, data were derived from a prospective, single-center, all-comer registry that included a cohort of consecutive patients with CAD who underwent PCI. We initially identified 788 patients who successfully underwent PCI for CAD and CT scans within six months of their index procedure. Subsequently, a series of exclusion criteria were applied: (1) patients who did not achieve successful PCI, (2) those who did not have available CT scans within one month before and after PCI, and (3) CT examinations lacking axial cross-sectional images at the L1 level. Following these exclusions, 475 patients were included in the present study, as shown in Figure 1. The study protocol followed the guidelines outlined in the Declaration of Helsinki and was approved by the Ethics Committee and Institutional Review Board at KUGH (2018GR0352).

### 2.2. Data Collection, Peri-Procedural Management, and Clinical Follow-Up

During index hospitalization for PCI, we conducted comprehensive patient interviews to capture demographic characteristics and assess cardiometabolic risk factors. We also obtained baseline laboratory results, which included an assessment of renal function, glycemic parameters, lipid profiles, and inflammatory markers. Renal function was quantified using the Cockcroft–Gault equation and presented as creatinine clearance [22]. The Rockwood Clinical Frailty Scale (CFS) was used to assess frailty levels during index admission. Frailty was identified by a CFS score ≥ 5, categorized as mild to moderate (CFS 5–6) and severe (CFS 7) functional disabilities [23]. Transthoracic echocardiography was performed to estimate the left ventricular ejection fraction using the modified Simpson biplane method. Significant valvular disease was defined as any moderate or severe valvular stenosis or regurgitation.

We enrolled patients who underwent successful PCI with stent implantation during the index procedure. Successful PCI was defined as meeting the angiographic criteria of the final angiogram showing residual stenosis of less than 30% and achieving thrombolysis in myocardial infarction grade 3 flow. The culprit or target lesion morphology was described according to the American College of Cardiology and American Heart Association lesion type classification [24]. Multivessel disease was defined as the presence of greater than 50% diameter stenosis in two or more major epicardial coronary arteries. Peri-procedural medications were prescribed in accordance with contemporary guidelines for managing CAD patients. The treatment duration and specific regimens for antithrombotic therapy, renin–angiotensin–aldosterone system blockers, statins, beta-blockers, and calcium channel blockers were tailored on a personalized basis depending on the attending physician’s discretion. The patients were scheduled to attend routine outpatient clinic visits within the first month after discharge and every 3–6 months thereafter. In cases in which patients missed their scheduled appointments, telephone interviews were conducted as an alternative method to evaluate adverse clinical events. The follow-up period was calculated from the date of index PCI procedure.

### 2.3. CT-Based Method of Skeletal Muscle Measurement

CT assessments were performed within one month of the index PCI procedure. The purpose of the CT scan was categorized as either elective or emergent based on clinically relevant indications. The CT protocols included the following five distinct categories: coronary CT, chest CT, lower-extremity CT angiography, abdomen–pelvis CT, and whole-body 18F-fluorodeoxyglucose-positron emission tomography-CT. Skeletal muscle area (SMA) measured at the level of the third lumbar vertebra (L3) has gained widespread acceptance as a reliable estimate of whole-body skeletal muscle mass and has been frequently utilized in prior studies [25,26,27]. However, cross-sectional images at the L3 level are frequently unavailable during the clinical assessment of CAD because these patients usually undergo CT protocols that primarily target the thoracic and upper lumbar vertebral regions. Therefore, the use of cross-sectional images from alternative vertebral levels is considered a potential option [28,29]. Recently, CT-based assessment of the SMA at the L1 level has been introduced and validated in various clinical conditions, including CAD and non-small-cell lung cancer [20,30]. Given this body of evidence, we specifically analyzed CT scans that included cross-sectional images at the L1 level to measure SMA.

**Figure 2:**
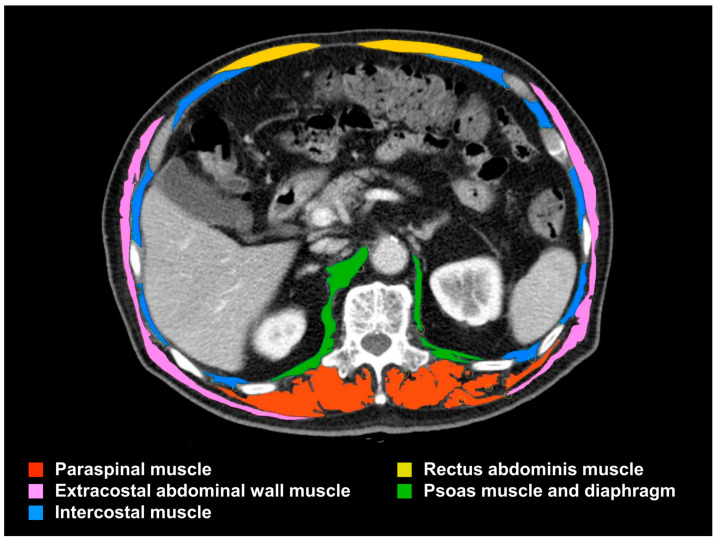
CT-based assessment of L1 skeletal muscle mass. The axial skeletal muscle area at the L1 level comprises five major components: paraspinal muscle, extracostal abdominal wall muscle, intercostal muscle, rectus abdominis muscle, and psoas muscle and diaphragm. CT, computed tomography; L1, first lumbar vertebra.

The SMA at the L1 level was measured as described previously [20]. In brief, CT Hounsfield unit ranges of −29 to 150 were considered skeletal muscle, and ranges of −190 to −30 were considered adipose tissue. The skeletal muscle measurements from the transverse cross-sectional CT image consisted of five major components, as shown in Figure 2: (1) the paraspinal muscle, (2) the extracostal abdominal wall muscle, (3) the intercostal muscle, (4) the rectus abdominis muscle, and (5) the psoas muscle and diaphragm. Two blinded observers independently measured the cross-sectional areas (cm^2^) of the five skeletal muscle components using a freehand module provided by the PACS workstation (G3 Infinitt PACS; Infinitt Healthcare, Seoul, Republic of Korea). The SMIs (cm^2^/m^2^) were determined by standardizing the measured cross-sectional SMA for height. L1 SMA and SMI measurements demonstrated excellent reproducibility and interobserver agreement in a previous study [20]. Subsequently, we stratified the entire study population into four groups based on sex-specific quartiles of L1 SMI to explore the potential existence of a dose-dependent relationship between skeletal muscle mass and adverse clinical outcomes. The sex-specific quartiles for L1 SMI were 30.00, 34.50, and 39.00 cm^2^/m^2^ for men and 24.50, 29.00, and 32.30 cm^2^/m^2^ for women. In the available cases, the skeletal muscles at the L3 level were measured as a composite of the abdominal wall, paraspinal, and psoas muscles (Figure S1) [17] to assess the correlation with the L1 skeletal muscle measurements.

### 2.4. Study Outcome Definitions

We conducted a clinical follow-up for all participants over a duration of up to three years. The primary endpoint in our study was the occurrence of all-cause mortality over 3-year period. The secondary endpoint was 3-year major adverse cardiovascular events (MACEs), defined as a composite of all-cause mortality, non-fatal myocardial infarction (MI), and repeat revascularization. Within the definition of mortality, we differentiated between those attributed to cardiovascular and non-cardiovascular causes, which were categorized as cardiac and non-cardiac deaths, respectively. MI was defined as a substantial increase in cardiac biomarkers along with concurrent symptoms or electrocardiogram findings suggestive of coronary ischemia. Repeat revascularization included all cases of clinically driven revascularization, which could involve either coronary artery bypass surgery or PCI and occurred after discharge from the initial procedure. For further clarification, repeat revascularization was specified in relation to the previously treated vessels or lesions. Finally, a landmark analysis at 1-year follow-up was performed to examine the prognostic significance of L1 SMI quartiles over both short- and long-term periods.

### 2.5. Statistical Analysis

Categorical variables were expressed as frequencies (percentages), and continuous variables were presented as means ± standard deviations or as medians with interquartile ranges. To compare groups across the L1 SMI quartiles, we used Pearson’s chi-square or Fisher’s exact test for categorical variables and one-way analysis of variance or the Kruskal–Wallis test for continuous variables. Pearson’s correlation coefficient was used to examine the correlations between various skeletal muscle measurements. The cumulative incidence rates for the primary and secondary endpoints were examined using the Kaplan–Meier method and compared using the log-rank test. Patients were censored at the time of the initial occurrence of the specific outcome measurement, with survival analysis conducted independently for both the individual outcome and the composite endpoints. Stepwise Cox-proportional hazards regression analysis was performed to assess the prognostic significance of the lower L1 SMI quartiles along with confounding factors. Four models were constructed by incorporating statistically significant or clinically relevant variables with the highest L1 SMI quartile used as the reference group. Statistical significance was defined as a *p*-value < 0.05. All analyses were two-tailed. All statistical analyses were performed using the Statistical Package for the Social Sciences (SPSS) software version 20.0 (SPSS-PC Inc., Chicago, IL, USA).

## 3. Results

### 3.1. Baseline Demographic Characteristics

The present study enrolled 475 patients who underwent successful coronary stent implantation for CAD with a median follow-up duration of 4.04 (1.25–3.00) years, and 82.3% of the patients completed the clinical visit at 3 years. The median time interval between the index PCI and CT imaging was −2.0 (−10.0–3.0) days, showing no significant difference between the L1 SMI quartile groups. Table 1 shows the baseline demographic characteristics of the study population. The average sex-specific L1 SMI values for the groups were as follows, displayed from the lowest to the highest L1 SMI quartiles: 25.98 ± 3.66, 32.49 ± 1.19, 36.92 ± 1.38, and 43.99 ± 3.82 cm^2^/m^2^ for men and 21.37 ± 2.56, 26.89 ± 1.34, 30.61 ± 0.92, and 35.76 ± 2.87 cm^2^/m^2^ for women (both *p* < 0.001). The lower L1 SMI quartile groups were associated with older age (72.41 ± 9.36 vs. 66.15 ± 9.70 vs. 63.57 ± 8.42 vs. 61.33 ± 9.86 years; *p* < 0.001), lower body mass index (21.58 ± 2.64 vs. 23.21 ± 2.32 vs. 24.36 ± 2.92 vs. 26.62 ± 2.58 kg/m^2^; *p* < 0.001), and higher burden of atherosclerotic disease for peripheral artery disease (16.9% vs. 8.6% vs. 5.3% vs. 7.3%; *p* = 0.013). Those with a lower L1 SMI exhibited worse renal and left ventricular systolic function (both *p* < 0.001), higher levels of inflammatory marker (*p* = 0.035) and frailty (*p* = 0.010). 

### 3.2. Correlation between L1 and L3 Skeletal Muscle Mass Measurements

Skeletal muscle measurements at both L1 and L3 levels were obtained from 68.6% (326/475) of the overall study participants. The L3 SMA and L3 SMI measurements showed significantly higher levels with increasing quartiles of L1 SMI (both *p* < 0.001). Notably, both SMA (R^2^ = 0.879, *p* < 0.001; Figure S2A) and SMI (R^2^ = 0.824, *p* < 0.001; Figure S2B) exhibited strong correlations between L1 and L3 measurements. Moreover, sex-specific correlative analyses of SMA and SMI consistently supported these findings (Figure S2C–E). These results suggest a robust correlation between skeletal muscle measurements at both the L1 and L3 levels. 

### 3.3. Procedural Characteristics and Peri-Procedural Medications

Table 2 presents an overview of procedural characteristics and peri-procedural medications. The distribution and number of treated vessels were well balanced across the L1 SMI quartiles. While the number of treated lesions (*p* = 0.049) and inserted stents (*p* = 0.043) marginally increased in the lower quartiles compared to those in the higher quartiles of L1 SMI, the overall disease extent, target/culprit lesion complexity, or coronary stent profiles, including the average diameter and total length, did not differ significantly between the L1 SMI quartile groups. The prescription patterns for secondary prevention medications after PCI were mostly consistent, except for higher statin use in the lower L1 SMI quartiles (89.4% vs. 97.3% vs. 88.7% vs. 80.6%; *p* < 0.001).

### 3.4. Three-Year Clinical Outcomes Based on the Sex-Specific L1 SMI Quartiles

**Figure 3:**
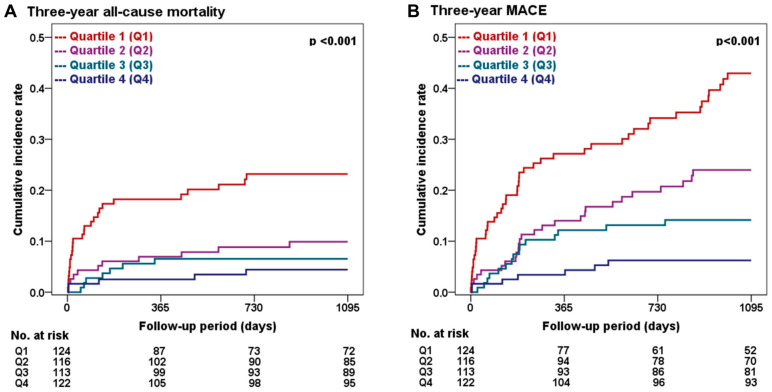
Kaplan–Meier survival curves for three-year clinical outcomes stratified by L1 SMI quartiles. The figure displays the three-year cumulative incidence rates of all-cause mortality (**A**) and MACE (**B**). MACE, major adverse cardiovascular event; Q1, quartile 1; Q2, quartile 2; Q3, quartile 3; Q4, quartile 4.

The three-year clinical outcomes of the groups categorized by sex-specific L1 SMI quartiles are presented in Table 3 and Figure 3. The primary endpoint of 3-year all-cause mortality showed a significantly higher incidence in the lower L1 SMI quartiles than in the higher quartiles (23.2% vs. 9.9% vs. 6.6% vs. 4.4%, *p* < 0.001; Figure 3A). This observed difference in all-cause mortality was primarily driven by a substantially higher rate of non-cardiac deaths in the lower L1 SMI group (17.0% vs. 5.5% vs. 5.7% vs. 1.8%, *p* < 0.001), implicating a potential association between non-cardiac medical comorbidities and sarcopenia. Table S1 shows the details of the specific etiologies of non-cardiac deaths. A trend toward an increase in cardiac deaths with lower L1 SMI values was observed, although the difference was not statistically significant (*p* = 0.081). The secondary composite endpoint of 3-year MACE showed a significantly higher incidence in the lower L1 SMI quartiles than in the higher quartiles (42.9% vs. 24.0% vs. 14.3% vs. 6.2%, *p* < 0.001; Figure 3B). More specifically, the cumulative incidence of individual composite endpoints, including non-fatal MI (8.7% vs. 3.0% vs. 2.0% vs. 2.6%, *p* = 0.038) and repeat revascularization (24.9% vs. 15.2% vs. 7.1% vs. 3.8%, *p* < 0.001), also significantly increased in the lower L1 SMI quartiles compared to those in the higher quartiles. Additional stratified analysis based on individual patients’ frailty status showed consistent trends for a higher incidence of 3-year all-cause mortality and MACE (Table S2).

### 3.5. Landmark Analysis at the One-Year Follow-Up

**Figure 4:**
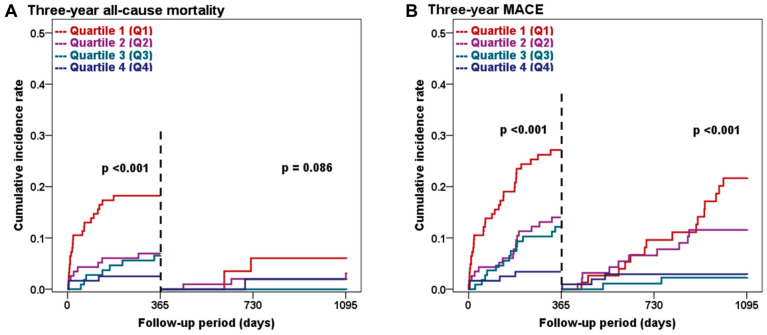
Landmark analysis at the one-year follow-up based on L1 SMI quartiles. The figure displays the cumulative incidence rates of all-cause mortality (**A**) and MACE (**B**) before and after the one-year follow-up. MACE, major adverse cardiovascular event; Q1, quartile 1; Q2, quartile 2; Q3, quartile 3; Q4, quartile 4.

The Kaplan–Meier survival curve showed a consistent increase in MACE incidence over the 3-year follow-up period, particularly in the lower L1 SMI quartiles (Figure 3B). Landmark analysis conducted at the one-year follow-up indicated a significant increase in the incidence of all-cause mortality and MACE during the first year following index PCI in the lower L1 SMI quartiles (both *p* < 0.001; Figure 4A,B). After the one-year time point, the difference in all-cause mortality based on L1 SMI quartiles showed a decreasing trend (*p* = 0.086; Figure 4A); however, the incidence of MACE continued to exhibit a significant difference (*p* < 0.001; Figure 4B) throughout the follow-up period, suggesting the potential of L1 SMI to predict both short- and long-term prognosis.

### 3.6. Stepwise Multivariate Analysis Based on the L1 SMI Quartiles

The results of the stepwise multivariate analysis are presented in Table 4. On univariate analyses, the lowest L1 SMI quartile showed a 6.07-fold higher risk of 3-year all-cause mortality (OR 6.07, 95% CI (2.33–15.7), *p* < 0.001) and an 8.45-fold higher risk of MACE (OR 8.45, 95% CI (3.81–18.7), *p* < 0.001) compared to the risks associated with the highest L1 SMI quartile. These associations remained consistent after adjusting for the constitutional factors of age, sex, and BMI (Model 2) as well as other significant confounding factors observed at index admission (Model 3). On multivariate analysis with full adjustment (Model 4), the L1 SMI quartiles continued to serve as significant predictors of both the primary and secondary endpoints. Specifically, the lowest L1 SMI quartile was associated with a 4.90-fold higher risk of 3-year all-cause mortality (OR 4.09, 95% CI (1.54–15.5), *p* = 0.007) and a 12.3-fold higher risk of MACE (OR 12.3, 95% CI (4.99–30.4), *p* < 0.001) compared to the highest L1 SMI quartile. Further details of this stepwise multivariate analysis are provided in Table S3.

## 4. Discussion

In this prospective all-comer registry study, we used CT-based measurements of L1 SMI and demonstrated a clear association between the future risk of 3-year all-cause mortality and MACE in patients who underwent successful PCI for CAD. Quantitative assessment of skeletal muscle mass using peri-procedural CT revealed a distinct dose–response relationship with future cardiovascular outcomes. This association was particularly pronounced for predicting 3-year all-cause mortality and MACE, underscoring the clinical significance of assessing skeletal muscle mass using L1 SMI. Notably, individuals in the lowest quartile of L1 SMI1 at baseline showed a 4.9-fold and 12.3-fold higher risk of 3-year all-cause mortality and MACE, respectively, than those in the highest quartile. Our stepwise multivariate analysis robustly confirmed the presence of a dose-response association between L1 SMI and 3-year adverse clinical outcomes. Furthermore, landmark analysis at one-year follow-up indicated that L1 SMI has the potential to predict both short- and long-term prognoses. Taken together, CT-based L1 SMI assessment may serve as a compelling quantitative prognostic marker for assessing future cardiovascular risk in CAD patients undergoing PCI.

### 4.1. CT-Based L1 SMI Measurement as a Quantitative Prognostic Marker for CAD

Compared to previously suggested indirect measurement modalities, CT-based skeletal muscle assessment has the key advantage of ensuring precise measurements with a wide range of clinical availability. The measurement of the SMA using CT scans for a specific anatomical region provides an accurate reflection of the muscle mass in a particular area [31]. Traditionally, SMA measured at the L3 level has been widely acknowledged as a reliable whole-body estimate of skeletal muscle mass [25,26,27]. Consequently, most prior studies focusing on sarcopenia have employed the SMA at the L3 level. However, in CAD patients, CT images are mostly confined to the thoracic and upper lumbar vertebral levels and require an alternative substitute for the L3 level. To overcome this limitation, the L1 level has been suggested as an alternative reference owing to its greater availability in chest and coronary CT scans [20,28,29,30]. Given this constraint in CAD patients, the present study revealed a robust correlation between CT-based skeletal muscle measurements obtained at both the L1 and L3 levels, suggesting its potential as an alternative option.

Our group recently validated the feasibility of CT-based L1 SMI measurements and demonstrated their prognostic significance in CAD patients undergoing successful PCI for the first time [20]. The CT-based L1 SMI measurement was highly reproducible, and we provided a sex-specific diagnostic threshold value of L1 SMI as 31.00 cm^2^/m^2^ in males and 25.00 cm^2^/m^2^ in females for defining sarcopenia. However, skeletal muscle mass and the diagnostic threshold for defining sarcopenia vary across races, ethnicities, and study populations [5,7,8,32]. Although employing cut-off values can improve clinical applicability, they may not sufficiently assess individual risk, especially when measurements are near the provided cut-off. Given the limitations of the previously suggested dichotomous threshold value, the present study comprehensively assessed the prognostic impact of L1 SMI to explore its potential dose-response relationship with future MACE. Intriguingly, L1 SMI exhibited a prominent quantitative effect on 3-year adverse clinical outcomes in the context of all-cause mortality and MACE. The lower quartiles of L1 SMI demonstrated a substantially higher risk of both life-threatening and less severe outcome measurements than those demonstrated by the higher quartiles. This suggests that alternative diagnostic threshold values may provide comparable outcome prediction results. Collectively, the present findings enhance our understanding of CT-based skeletal muscle mass measurements and broaden our perspective depending on specific diagnostic threshold values.

### 4.2. Biological Mechanism Linking Sarcopenia and Future Cardiovascular Risk

Sarcopenia is a clinical condition with multifactorial causes and associated risk factors. Sarcopenia often coexists with chronic medical conditions, including malignancies, endocrine disorders, chronic kidney diseases, respiratory diseases, autoimmune disorders, and ASCVD [11,33]. Several factors, such as low physical activity, malnutrition, insulin resistance, oxidative stress, systemic inflammation, and hormonal dysregulation have been suggested to be involved in the simultaneous presence of ASCVD and sarcopenia [11,34]. In the present study population, the serum inflammatory marker high-sensitivity C-reactive protein increased substantially and the renal function decreased notably, as assessed by creatinine clearance, among individuals in the lower quartiles of L1 SMI compared to those in the higher quartiles at baseline. These findings support the mechanistic link between sarcopenia and underlying cardiometabolic risk factors. Furthermore, sarcopenia can result in poor cardiopulmonary function and contribute to a higher prevalence of cardiometabolic risk factors [35,36]. Mechanistically, muscle cells play a crucial role in the cardiovascular system by releasing various bioactive molecules called myokines, which have been suggested to have beneficial effects on cardiovascular heath [11,37]. Loss of skeletal muscle mass can compromise the endocrine function and contribute to adverse cardiovascular outcomes [38]. For instance, a reduction in skeletal muscle mass may lead to increased insulin resistance and alterations in glucose metabolism, both of which can negatively affect cardiovascular events [39,40]. Both a reduction in the number of muscle cells and deterioration in their endocrine function may have played a crucial role in the unfavorable clinical outcomes in individuals with low L1 SMI in the present study. Moreover, individuals in lower L1 SMI quartiles tended to have higher frailty levels. This increased frailty was consistently associated with a higher incidence of 3-year all-cause mortality and MACE. Hence, the integration of CT-based skeletal muscle data with clinical frailty scales could significantly enhance our understanding of the mechanistic interconnections between frailty and skeletal muscle mass and their implications for cardiovascular health.

### 4.3. Clinical and Therapeutic Implication of Assessing Sarcopenia in CAD

The current study’s findings suggest that assessing skeletal muscle mass in patients undergoing PCI during the periprocedural period could offer complementary insights along with conventional risk factors. More specifically, the significantly increased risk of non-cardiac mortality implies that sarcopenia is a complex clinical condition frequently associated with various underlying medical comorbidities. Therefore, it may be relevant to conduct a comprehensive assessment of coexisting modifiable medical factors in individuals with sarcopenia to improve overall clinical outcomes. Furthermore, addressing sarcopenia could offer an additional theoretical basis for secondary prevention strategies, including cardiac rehabilitation and non-pharmacological physical intervention in CAD patients. Contemporary guidelines for the management of CAD strongly recommend multidisciplinary cardiovascular rehabilitation as a Class I recommendation based on its favorable impact on overall health [41,42]. Increased physical activity and improved cardiac fitness through cardiovascular rehabilitation may effectively prevent further decline in skeletal muscle mass and consequently result in better clinical outcomes. Moreover, the assessment of skeletal muscle mass using L1 SMI could help identify a specific subgroup of patients who could obtain maximal benefits from cardiovascular rehabilitation, as suggested by the potential dose–response relationship demonstrated in the present study. Finally, the landmark analysis at the one-year time point demonstrated the prognostic significance of L1 SMI, both within and beyond the first year following the index PCI procedure. This finding suggests that evaluating sarcopenia during this period could offer valuable insights into both the short- and long-term prognoses.

### 4.4. Limitation and Future Perspectives

First, this study was based on a secondary analysis of non-randomized all-comer registry data. Although we implemented a multiple-stepwise Cox proportional hazards regression analysis, we must acknowledge that residual confounding factors might have influenced the clinical outcomes. Second, measurement of skeletal muscle mass was not the main objective of the CT scans performed in our study. Although our previous study demonstrated that L1 SMI measurements from different CT scan protocols are highly reproducible, future prospective studies should be conducted using a uniform and standardized image acquisition protocol to ensure a more precise and consistent assessment. Additionally, the integration of an artificial intelligence-assisted automated quantification algorithm has the potential to improve the clinical applicability of CT-based skeletal muscle assessments [29]. Third, this study primarily focused on skeletal muscle as a representative body composition for predicting future MACE. In addition to skeletal muscle, various body components, such as visceral fat, have been recognized as significant contributors to adverse clinical outcomes in ASCVD patients [43,44]. Future studies that include additional body composition parameters may offer opportunities to evaluate muscle quality, specifically by focusing on myosteatosis [45,46]. This approach may lead to a more comprehensive understanding of the mechanistic interplay between sarcopenia and cardiometabolic risk. Fourth, the sample size of our study (475 patients) was limited, providing strong evidence to support the main study findings. The results of the present study should be considered hypothesis-generating, and future studies with larger sample sizes are needed to ensure sufficient statistical power. Moreover, to substantiate the clinical relevance of our findings, a well-designed prospective randomized study incorporating appropriate therapeutic interventions is required. Finally, muscle strength assessments were not routinely performed in our study. Contemporary international guidelines and expert consensus emphasize both muscle strength and quantity assessments for diagnosing sarcopenia [6,7,8]. Although skeletal muscle mass is consistently required for diagnosing sarcopenia, future studies should include both muscle strength and quantity information for a comprehensive assessment.

## 5. Conclusions

The skeletal muscle mass, as assessed using L1 SMI, demonstrated a distinct dose-dependent relationship with future adverse clinical outcomes in CAD patients who underwent successful PCI. Integrating CT-based sarcopenia assessment into routine daily clinical practice has the potential to enhance risk stratification and provide valuable prognostic insights for guiding therapeutic decisions in patients with established ASCVD.

## Figures and Tables

**Table 1:** Baseline clinical characteristics.

	L1 SMI Q1 (n = 124)	L1 SMI Q2 (n = 116)	L1 SMI Q3 (n = 112)	L1 SMI Q4 (n = 123)	*p*-Value
Demographic feature					
Age (years)	72.41 ± 9.36	66.15 ± 9.70	63.57 ± 8.42	61.33 ± 9.86	<0.001
Gender (male)	88 (70.9)	78 (67.2)	78 (69.6)	86 (69.9)	0.937
BMI (kg/m^2^)	21.58 ± 2.64	23.21 ± 2.32	24.36 ± 2.92	26.62 ± 2.58	<0.001
Clinical presentation					
Myocardial infarction	38 (30.6)	31 (26.7)	43 (38.3)	31 (25.2)	0.127
Unstable angina	32 (25.8)	39 (33.6)	28 (25.0)	47 (38.2)	0.078
Stable angina	45 (36.2)	41 (35.3)	37 (33.0)	40 (32.5)	0.913
Past medical history					
Previous CAD	78 (62.9)	73 (62.9)	69 (61.6)	88 (71.5)	0.344
Hypertension	61 (49.1)	55 (47.4)	44 (39.2)	53 (43.0)	0.422
Diabetes	14 (11.2)	23 (19.8)	23 (20.5)	23 (18.6)	0.204
Diabetes with insulin therapy	23 (18.5)	27 (23.3)	17 (15.2)	20 (16.3)	0.392
Dyslipidemia	11 (8.8)	9 (7.7)	7 (6.2)	13 (10.5)	0.678
Cerebrovascular accident	27 (21.7)	17 (14.6)	15 (13.3)	21 (17)	0.320
Peripheral artery disease	21 (16.9)	10 (8.6)	6 (5.3)	9 (7.3)	0.013
Heart failure					
LVEF < 50%	41 (33.1)	27 (23.3)	26 (23.2)	16 (13.0)	0.003
LVEF < 40%	20 (16.1)	13 (11.2)	11 (9.8)	8 (6.5)	0.109
Atrial fibrillation	10 (8.1)	2 (1.7)	6 (5.4)	7 (5.7)	0.153
Significant valvular disease	6 (4.8)	2 (1.7)	1 (0.9)	3 (2.4)	0.285
Chronic kidney disease (stage ≥ 3)	68 (54.8)	38 (32.8)	28 (25.0)	21 (17.1)	<0.001
Renal replacement therapy	8 (6.5)	3 (2.6)	1 (0.9)	4 (3.3)	0.114
Previous malignancy	11 (8.8)	8 (6.8)	5 (4.4)	8 (6.5)	0.607
Current smoker	41 (33.0)	41 (35.3)	37 (33.0)	44 (35.7)	0.953
Frailty (CFS ≥ 5)	44 (35.5)	31 (26.7)	24 (21.4)	22 (17.9)	0.010
Mild to moderate (CFS 5–6)	33 (26.6)	29 (25.0)	21 (18.8)	22 (17.9)	
Severe (CFS 7)	2 (1.7)	3 (2.7)	0 (0.0)	16 (3.4)	
Laboratory data					
Total cholesterol (mg/dL)	158.0 (128.2–185.7)	156.0 (129.0–187.0)	163.0 (136.0–198.0)	173.5 (144.2–203.0)	0.055
LDL-c (mg/dL)	104.0 (77.5–125)	94.0 (75.8–128.0)	95.0 (74.0–130.0)	110.5 (85.5–140.7)	0.184
hs-CRP (mg/L)	6.0 (1.0–17.5)	3.0 (1.0–12.3)	2.0 (1.0–9.0)	2.0 (1.0–7.5)	0.035
HbA1c (%)	6.40 ± 1.46	6.34 ± 1.70	6.61 ± 1.92	6.72 ± 1.63	0.301
Serum creatinine (mg/dL)	1.46 ± 2.17	1.19 ± 1.32	1.29 ± 2.10	1.16 ± 1.40	0.573
CrCl (mL/min)	57.99 ± 27.27	70.37 ± 29.24	78.98 ± 30.42	88.07 ± 30.84	<0.001
LVEF (%)	50.17 ± 11.47	53.02 ± 11.10	53.56 ± 10.15	55.84 ± 6.98	<0.001
CT scan information					
Average days from PCI to CT scan	−1.89 ± 11.72	−3.58 ± 10.79	−3.52 ± 11.02	−4.40 ± 13.19	0.407
L1 SMA (cm^2^)	65.40 ± 15.34	81.69 ± 15.06	92.93 ± 16.06	111.33 ± 22.23	<0.001
Male	72.28 ± 12.21	91.30 ± 6.57	102.37 ± 7.95	123.93 ± 11.88	<0.001
Female	48.57 ± 6.82	61.96 ± 4.50	70.98 ± 3.93	82.38 ± 9.81	<0.001
L1 SMI (cm^2^/m^2^)	24.64 ± 3.97	30.65 ± 2.91	35.02 ± 3.17	41.49 ± 5.20	<0.001
Male	25.98 ± 3.66	32.49 ± 1.19	36.92 ± 1.38	43.99 ± 3.82	<0.001
Female	21.37 ± 2.56	26.89 ± 1.34	30.61 ± 0.92	35.76 ± 2.87	<0.001
Available for L3 assessment	93 (75.0)	74 (63.8)	76 (67.9)	83 (67.5)	0.295
L3 SMA (cm^2^)	78.96 ± 17.49	102.93 ± 20.76	114.75 ± 23.74	134.78 ± 28.75	<0.001
Male	87.28 ± 15.19	114.69 ± 16.73	127.99 ± 17.30	149.96 ± 20.00	<0.001
Female	63.46 ± 8.80	82.93 ± 9.80	89.03 ± 10.35	101.52 ± 14.80	<0.001
L3 SMI (cm^2^/m^2^)	29.96 ± 4.77	38.84 ± 4.71	43.38 ± 5.78	50.44 ± 7.22	<0.001
Male	31.38 ± 4.80	40.55 ± 4.99	45.97 ± 5.16	53.04 ± 6.63	<0.001
Female	27.73 ± 3.68	36.07 ± 3.68	38.29 ± 3.59	44.37 ± 5.02	<0.001

Data are expressed as n (%), mean ± SD or median (interquartile range). BMI, body mass index; CAD, coronary artery disease; CFS; clinical frailty scale; CrCl, creatinine clearance; CT, computed tomography; hs-CRP, high sensitivity C-reactive protein; L1, first lumbar vertebra; L3, third lumbar vertebra; LDL-c, low density lipoprotein cholesterol; LVEF, left ventricular ejection fraction; PET, positron-emission tomography; PCI, percutaneous coronary intervention; SMA, skeletal muscle area; SMI, skeletal muscle index; Q1, quartile 1; Q2, quartile 2; Q3, quartile 3; Q4, quartile 4.

**Table 2:** Procedural characteristics.

	L1 SMI Q1 (n = 124)	L1 SMI Q2 (n = 116)	L1 SMI Q3 (n = 112)	L1 SMI Q4 (n = 123)	*p*-Value
PCI procedural profiles					
Number of treated lesions	1.89 ± 1.14	1.71 ± 0.98	1.53 ± 0.90	1.71 ± 1.04	0.049
Number of treated vessels	1.37 ± 0.63	1.27 ± 0.50	1.24 ± 0.47	1.29 ± 0.59	0.280
Treated vessels					
Left main	6 (4.8)	3 (2.5)	5 (4.4)	3 (2.4)	0.677
LAD	77 (62.0)	70 (60.3)	61 (54.4)	70 (56.9)	0.636
LCX	36 (29.0)	21 (18.1)	36 (32.1)	41 (33.3)	0.039
RCA	46 (37.0)	53 (45.6)	39 (34.8)	43 (34.9)	0.271
Lesion type B2C	115 (92.7)	110 (94.8)	107 (95.5)	115 (93.4)	0.796
Multivessel disease	31 (25.0)	29 (25.0)	31 (27.6)	26 (21.1)	0.709
Left main disease	9 (7.2)	9 (7.7)	11 (9.8)	8 (6.5)	0.807
Diffuse lesion (>30 mm)	46 (37)	47 (40.5)	49 (43.7)	44 (35.7)	0.595
Small vessel disease (<2.25 mm)	7 (5.6)	7 (6.0)	11 (9.8)	9 (7.3)	0.605
Intravascular imaging	13 (10.5)	9 (7.8)	13 (12.6)	10 (8.1)	0.706
Number of inserted stents	1.85 ± 1.01	1.70 ± 0.96	1.50 ± 0.80	1.68 ± 0.97	0.043
Average stent diameter (mm)	2.90 ± 0.39	3.01 ± 0.46	3.02 ± 0.37	2.98 ± 0.46	0.128
Total stent length (mm)	43.67 ± 27.48	40.14 ± 27.69	36.49 ± 23.01	39.26 ± 28.28	0.210
Bare metal stents	2 (1.6)	5 (4.3)	2 (1.7)	1 (0.8)	0.361
Drug eluting stents	122 (98.3)	113 (97.4)	110 (98.2)	123 (100.0)	0.404
1st generation	25 (20.1)	28 (24.1)	23 (20.5)	17 (13.8)	0.239
2nd generation	97 (78.2)	85 (73.2)	87 (77.6)	106 (86.1)	0.100
Post-procedural medication					
Aspirin	112 (90.3)	106 (91.3)	105 (93.7)	113 (91.8)	0.813
Clopidogrel	101 (81.4)	104 (89.6)	102 (91.0)	110 (89.4)	0.091
Oral anticoagulants	5 (4.0)	1 (0.9)	6 (5.4)	5 (4.1)	0.250
RAS blockers	71 (57.2)	75 (64.6)	70 (62.5)	80 (65.0)	0.570
Statins	110 (89.4)	109 (97.3)	103 (88.7)	100 (80.6)	<0.001
Beta blockers	62 (50.0)	53 (45.6)	56 (50.0)	58 (47.1)	0.884
Calcium channel blockers	40 (32.2)	38 (32.7)	34 (30.3)	38 (30.8)	0.977

Data are expressed as n (%), mean ± SD or median (interquartile range). LAD, left anterior descending artery; LCX, left circumflex artery; L1, first lumbar vertebra; PCI, percutaneous coronary intervention; RAS, renin-angiotensin-aldosterone system; RCA, right coronary artery; SMI, skeletal muscle index; Q1, quartile 1; Q2, quartile 2; Q3, quartile 3; Q4, quartile 4.

**Table 3:** Three-year clinical outcomes based on L1 SMI quartiles.

	L1 SMI Q1 (n = 124)	L1 SMI Q2 (n = 116)	L1 SMI Q3 (n = 112)	L1 SMI Q4 (n = 123)	Log-Rank *p*-Value
All-cause mortality	27 (23.2)	11 (9.9)	7 (6.6)	5 (4.4)	<0.001
Cardiac death	8 (7.4)	5 (4.6)	1 (1.0)	3 (2.6)	0.081
Non-cardiac death	19 (17.0)	6 (5.5)	6 (5.7)	2 (1.8)	<0.001
Non-fatal MI	9 (8.7)	3 (3.0)	2 (2.0)	3 (2.6)	0.038
STEMI	5 (4.4)	1 (1.2)	1 (1.0)	2 (1.8)	0.152
Non-STEMI	4 (4.5)	2 (1.8)	1 (1.0)	1 (0.8)	0.301
Repeat revascularization	20 (24.9)	15 (15.2)	7 (7.1)	4 (3.8)	<0.001
TVR	16 (20.3)	11 (11.1)	6 (6.2)	3 (2.8)	0.001
non-TVR	6 (8.1)	5 (5.9)	3 (3.1)	1 (1.0)	0.114
MACE	47 (42.9)	26 (24.0)	15 (14.3)	7 (6.2)	<0.001

Data are expressed as incidence (%). L1, first lumbar vertebra; MACE, major adverse cardiovascular event; MI, myocardial infarction; NSTEMI, non-ST-elevation myocardial infarction; Q1, quartile 1; Q2, quartile 2; Q3, quartile 3; Q4, quartile 4; TVR, target vessel revascularization; SMI, skeletal muscle index; STEMI, ST-elevation myocardial infarction.

**Table 4:** Stepwise multivariate analysis for 3-year clinical outcomes.

	3-Year All-Cause Mortality		3-Year MACE	
	OR (95% CI)	*p*-Value	OR (95% CI)	*p*-Value
Model 1 ^a^				
L1 SMI quartiles		<0.001 (for trend)		<0.001 (for trend)
Quartile 4	1.00 (reference)		1.00 (reference)	
Quartile 3	1.52 (0.48–4.81)	0.468	2.41 (0.98–5.91)	0.054
Quartile 2	2.32 (0.80–6.68)	0.118	4.13 (1.79–9.52)	0.001
Quartile 1	6.07 (2.33–15.7)	<0.001	8.45 (3.81–18.7)	<0.001
Model 2 ^b^				
L1 SMI quartiles		0.007 (for trend)		<0.001 (for trend)
Quartile 4	1.00 (reference)		1.00 (reference)	
Quartile 3	1.71 (0.52–5.56)	0.370	3.17 (1.28–7.86)	0.013
Quartile 2	2.20 (0.72–6.74)	0.164	5.93 (2.47–14.2)	<0.001
Quartile 1	5.62 (1.77–17.8)	0.003	15.5 (6.28–38.4)	<0.001
Model 3 ^c^				
L1 SMI quartiles		0.030 (for trend)		<0.001 (for trend)
Quartile 4	1.00 (reference)		1.00 (reference)	
Quartile 3	1.76 (0.54–5.71)	0.342	3.09 (1.25–7.65)	0.014
Quartile 2	2.32 (0.76–7.08)	0.139	5.95 (2.49–14.2)	<0.001
Quartile 1	4.93 (1.54–15.7)	0.007	12.7 (5.13–31.6)	<0.001
Model 4 ^d^				
L1 SMI quartiles		0.032 (for trend)		<0.001 (for trend)
Quartile 4	Reference		Reference	
Quartile 3	1.83 (0.56–5.97)	0.315	3.23 (1.29–8.07)	0.012
Quartile 2	2.25 (0.74–6.79)	0.149	5.54 (2.31–13.2)	<0.001
Quartile 1	4.90 (1.54–15.5)	0.007	12.3 (4.99–30.4)	<0.001

^a^ Model 1 represents univariate analysis; ^b^ Model 2 was adjusted for age, sex, and body mass index; ^c^ Model 3 included further adjustments for left ventricular ejection fraction < 50%, creatinine clearance < 60 mL/min, prescription of statins, number of treated lesions, and number of inserted stents; ^d^ Model 4 further incorporated adjustments for stent implantation for myocardial infarction, hypertension, diabetes, previous malignancy, multivessel disease, and implantation of 2nd-generation drug-eluting stents. CI, confidence interval; L1, first lumbar vertebra; MACE, major adverse cardiovascular event; OR, odds ratio; SMI, skeletal muscle index.

## Data Availability

The corresponding author has complete access to the original study data, and anonymized data will be made available upon request with a reasonable purpose.

## Supplementary Materials

The following supporting information can be downloaded at: https://www.mdpi.com/article/10.3390/jcm12237483/s1, Table S1: Detailed etiologies of non-cardiac death in the study population. Table S2: Three-year clinical outcomes based on the frailty levels. Table S3: Complete dataset of stepwise multivariate analysis for 3-year clinical outcomes. Figure S1: Representative CT-based L3 skeletal muscle measurement. Figure S2: Correlation between L1 and L3 skeletal muscle measurements.
