# Supplementary material for: Quantitative Association between Computed-Tomography-Based L1 Skeletal Muscle Indices and Major Adverse Clinical Events Following Percutaneous Coronary Intervention
Source: J Clin Med. 2023 Dec 3;12(23):7483. doi: 10.3390/jcm12237483 (PMC10707591; doi:10.3390/jcm12237483)
Supplement: Supplementary file 1 [file jcm-12-07483-s001.zip › jcm-2723197-supplementary.pdf]

Supplementary Data

Quantitative Association Between Computed Tomography-based L1 Skeletal Muscle Indices and Major Adverse Clinical Events Following Percutaneous Coronary Intervention

Eun Jin Park[a, †], So Yeon Park[b, †], Jaeho Kang[a], Wonsang Chu[a], and Dong Oh Kang[a, **]

[a] Cardiovascular Center, Department of Internal Medicine, Korea University Guro Hospital, Korea University College of Medicine, Seoul, Republic of Korea

[b] Healthcare Center, Department of Radiology, Gangnam Severance Hospital, Seoul, Republic of Korea

[†] These authors contributed equally to this work

Supplementary Tables

Table S1. Detailed etiologies of non-cardiac death in the study population

	**Total population (n=475)**	**L1 SMI Q1 (n=124)**	**L1 SMI Q2 (n=116)**	**L1 SMI Q3 (n=112)**	**L1 SMI Q4 (n=123)**
Non-cardiac death	33 (6.9)	19 (15.3)	6 (5.2)	6 (5.4)	2 (1.6)
Infectious disease	16 (3.4)	11 (8.9)	2 (1.7)	2 (1.8)	1 (0.8)
Intracranial hemorrhage	6 (1.3)	3 (2.4)	1 (0.9)	1 (0.9)	1 (0.8)
Gastrointestinal bleeding	2 (0.4)	1 (0.8)	1 (0.9)	0 (0.0)	0 (0.0)
Respiratory failure	5 (1.1)	2 (1.6)	1 (0.9)	2 (1.8)	0 (0.0)
Malignancy	3 (0.6)	1 (0.8)	1 (0.9)	1 (0.9)	0 (0.0)
Acute kidney injury	1 (0.2)	1 (0.8)	0 (0.0)	0 (0.0)	0 (0.0)

Data are expressed as n (%). L1, first lumbar vertebra; Q1, quartile 1; Q2, quartile 2; Q3, quartile 3; Q4, quartile 4; SMI, skeletal muscle index.

Table S2. Three-year clinical outcomes based on the frailty levels

	Non-frail (n=354)	Mild to moderate frailty (n=105)	Severe frailty (n=116)	Log-rank P-value
All-cause mortality	22 (6.4)	19 (19.1)	9 (58.3)	<0.001
Cardiac death	7 (2.0)	7 (7.4)	3 (26.2)	<0.001
Non-cardiac death	15 (4.5)	12 (12.6)	6 (43.3)	<0.001
Non-fatal MI	10 (3.0)	5 (5.5)	2 (20.9)	0.070
Repeat revascularization	36 (12.1)	10 (13.3)	0 (0.0)	0.504
MACE	58 (17.8)	28 (29.1)	9 (58.3)	<0.001

Data are expressed as incidence (%). Frailty status was assessed using the Rockwood Clinical Frailty Scale (CFS), categorized as non-frail (CFS <5), mild to moderate frailty (CFS 5-6), and severe frailty (CFS 7). MACE, major adverse cardiovascular event; MI, myocardial infarction.

Table S3. Complete dataset of stepwise multivariate analysis for 3-year clinical outcomes

	3-year all-cause mortality		3-year MACE	
	OR (95% CI)	P-value	OR (95% CI)	P-value
**Model 2**				
L1 SMI quartiles		0.007 (for trend)		<0.001 (for trend)
Quartile 4	Reference		Reference	
Quartile 3	1.71 (0.52–5.56)	0.370	3.17 (1.28–7.86)	0.013
Quartile 2	2.20 (0.72–6.74)	0.164	5.93 (2.47–14.2)	<0.001
Quartile 1	5.62 (1.77–17.8)	0.003	15.5 (6.28–38.4)	<0.001
Age >65 years	0.93 (0.51–1.68)	0.820	0.89 (0.57–1.39)	0.617
Gender (male)	4.25 (1.74–10.3)	0.001	1.38 (0.86–2.24)	0.179
BMI (per 1.0 kg/m^2 increase)	1.04 (0.94–1.16)	0.380	1.13 (1.05–1.21)	0.001
**Model 3**				
L1 SMI quartiles		0.030 (for trend)		<0.001 (for trend)
Quartile 4	Reference		Reference	
Quartile 3	1.76 (0.54–5.71)	0.342	3.09 (1.25–7.65)	0.014
Quartile 2	2.32 (0.76–7.08)	0.139	5.95 (2.49–14.2)	<0.001
Quartile 1	4.93 (1.54–15.7)	0.007	12.7 (5.13–31.6)	<0.001
Age >65 years	1.14 (0.62–2.10)	0.654	1.06 (0.67–1.68)	0.793
Gender (male)	3.32 (1.33–8.23)	0.010	1.12 (0.68–1.84)	0.642
BMI (per 1.0 kg/m^2 increase)	1.09 (0.98–1.21)	0.107	1.15 (1.07–1.24)	<0.001

LVEF <50%	1.43 (0.78–2.59)	0.238	1.40 (0.90–2.18)	0.129
CrCl <60 mL/min	1.85 (0.97–3.51)	0.059	1.79 (1.13–2.86)	0.013
Statin prescription	0.46 (0.23–0.90)	0.024	0.56 (0.32–0.97)	0.039
Number of treated lesions (per 1.0 increase)	1.03 (0.20–5.16)	0.967	0.74 (0.24–2.26)	0.606
**Model 4**				
L1 SMI quartiles		0.032 (for trend)		<0.001 (for trend)
Quartile 4	Reference		Reference	
Quartile 3	1.83 (0.56–5.97)	0.315	3.23 (1.29–8.07)	0.012
Quartile 2	2.25 (0.74–6.79)	0.149	5.54 (2.31–13.2)	<0.001
Quartile 1	4.90 (1.54–15.5)	0.007	12.3 (4.99–30.4)	<0.001
Age >65 years	1.06 (0.57–1.97)	0.834	1.00 (0.63–1.59)	0.986
Gender (male)	3.25 (1.29–8.19)	0.012	1.07 (0.64–1.78)	0.785
BMI (per 1.0 kg/m^2 increase)	1.07 (0.96–1.20)	0.182	1.14 (1.06–1.23)	<0.001
LVEF <50%	1.29 (0.68–2.45)	0.431	1.31 (0.81–2.11)	0.264
CrCl <60 mL/min	1.67 (0.87–3.23)	0.121	1.70 (1.04–2.75)	0.031
Statin prescription	0.45 (0.22–0.92)	0.031	0.58 (0.33–1.02)	0.060
Number of treated lesions (per 1.0 increase)	0.91 (0.18–4.64)	0.915	0.59 (0.18–1.88)	0.379
Number of inserted stents (per 1.0 increase)	1.58 (0.25–9.74)	0.620	2.32 (0.63–8.49)	0.200

PCI for myocardial infarction	0.65 (0.34–1.24)	0.197	0.78 (0.48–1.27)	0.327
Hypertension	0.91 (0.47–1.76)	0.799	0.88 (0.55–1.41)	0.612
Diabetes	2.00 (1.07–3.74)	0.029	1.76 (1.13–2.74)	0.012
Previous malignancy	1.73 (0.70–4.25)	0.232	1.15 (0.53–2.49)	0.719
Multivessel disease	0.55 (0.23–1.32)	0.187	0.86 (0.47–1.57)	0.631
Second generation DES	0.90 (0.46–1.73)	0.754	0.75 (0.47–1.19)	0.227

BMI, body mass index; CI, confidence interval; CrCl, creatinine clearance; DES, drug eluting stents; L1, first lumbar vertebra; LVEF, left ventricular ejection fraction; MACE, major adverse cardiovascular event; OR, odds ratio; PCI, percutaneous coronary intervention; SMI, skeletal muscle index

Supplementary Figures

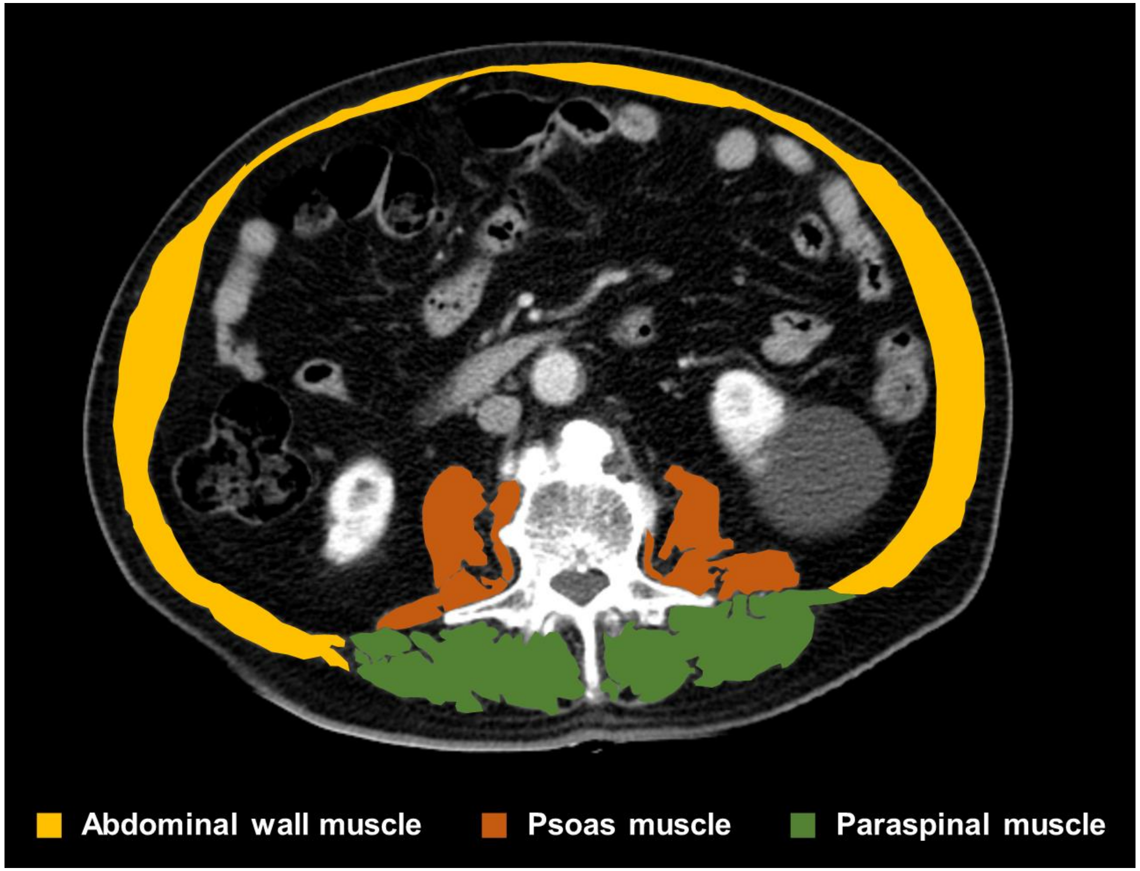

Figure S1. Representative CT-based L3 skeletal muscle measurement

Measurement of skeletal muscles at the L3 level consisted of the abdominal wall, psoas, and paraspinal muscles. CT, computed tromography; L3, third lumbar vertebra

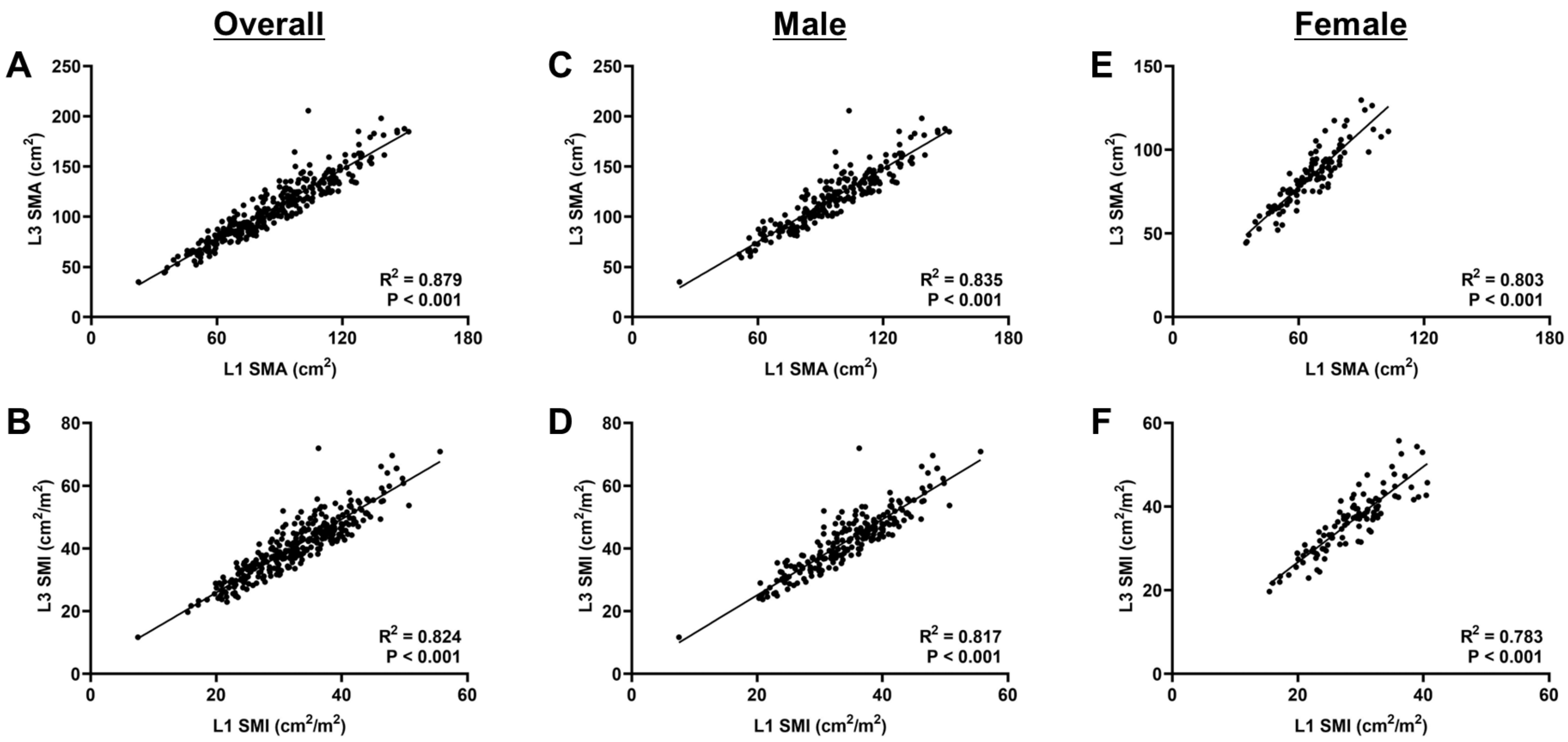

Figure S2. Correlation between L1 and L3 skeletal muscle measurements

Correlative analysis of L1 versus L3 SMA and SMI in the overall population **(A, B)**, male patients **(C, D)**, and female patients **(E, F)**. L1, first lumbar vertebra; L3, third lumbar vertebra; SMA, skeletal muscle area; SMI, skeletal muscle index.
